# Psychological Momentum—A Key to Continued Success

**DOI:** 10.3389/fpsyg.2016.01328

**Published:** 2016-08-31

**Authors:** Seppo E. Iso-Ahola, Charles O. Dotson

**Affiliations:** Department of Kinesiology, University of Maryland, College ParkCollege Park, MD, USA

**Keywords:** psychological momentum, performance, success, automaticity, consciousness

## Abstract

One of the most fundamental characteristics about humans is their desire for success, especially in highly competitive societies. What does it take to be successful? Is success simply a matter of better performance, and if so, what specifically is it about performance that determines success? A long research tradition suggests that psychological momentum (PM) plays a critical role in goal pursuit and achievement. Accordingly, sequential runs of success are an essential feature of high levels of performance, meaning that better performers perceive and experience momentum of success more frequently, ride it as long as they can, and as a result, become more successful in the end. Theoretically, momentum is a principle vehicle of performance that will significantly augment future success and facilitate goal achievement. Consequently, an overall performance consists of occurrences of momentum that vary in frequency and duration. The higher the frequency and the higher the duration, the more likely is success. Research suggests that the main psychological processes that underpin momentum effects are confidence, perceived competence, and internal (ability-skill) attributions. Based upon related research, it is hypothesized that PM starts as a conscious process but subsequently becomes a major facilitator of nonconscious automatic execution of human behavior and performance.

## Introduction

Psychological momentum (PM) is conceptualized as a perceptual phenomenon that changes human behavior and performance. It is “experienced as a psychological force in which several factors or qualities converge in a synergistic way to enable one to perform at a level not ordinarily possible” (Iso-Ahola and Dotson, 2014, p. 20). Tennis pros, for example, can grow more confident after winning a game. But if such a single successful performance does not lead to an altered state of mind according to which they see success becoming a real possibility and sense things going inevitably their way, they will not have PM that can be ridden to further success. Importantly, PM explains variations in performance (Hubbard, 2015a,b), influences elite performers' actions (Attali, 2013) and coaches' behavior (Raab et al., 2012), and impacts the behavior and experience of spectators of sporting events (Markman and Guenther, 2007).

The phenomenon is *ubiquitous*, ranging from doing household chores to trading stocks, driving in traffic, winning Presidential primaries, and beating opponents in sports. When NBA star Stephen Curry gains momentum in making baskets, he is no different than a person who gets on a roll doing household chores–vacuuming room after room, tidying, and dusting (i.e., beating the opponent of the dirty house). PM, of course, works both ways. When stock traders lose money one trade after another, they spiral into negative momentum and worsening performance. This examination of research, however, focuses on positive momentum, its antecedents and consequences, because people generally aim to gain positive momentum rather than avoid negative momentum (Briki et al., 2013; Iso-Ahola and Dotson, 2014; Hubbard, 2015a,b).

**Figure 1:**
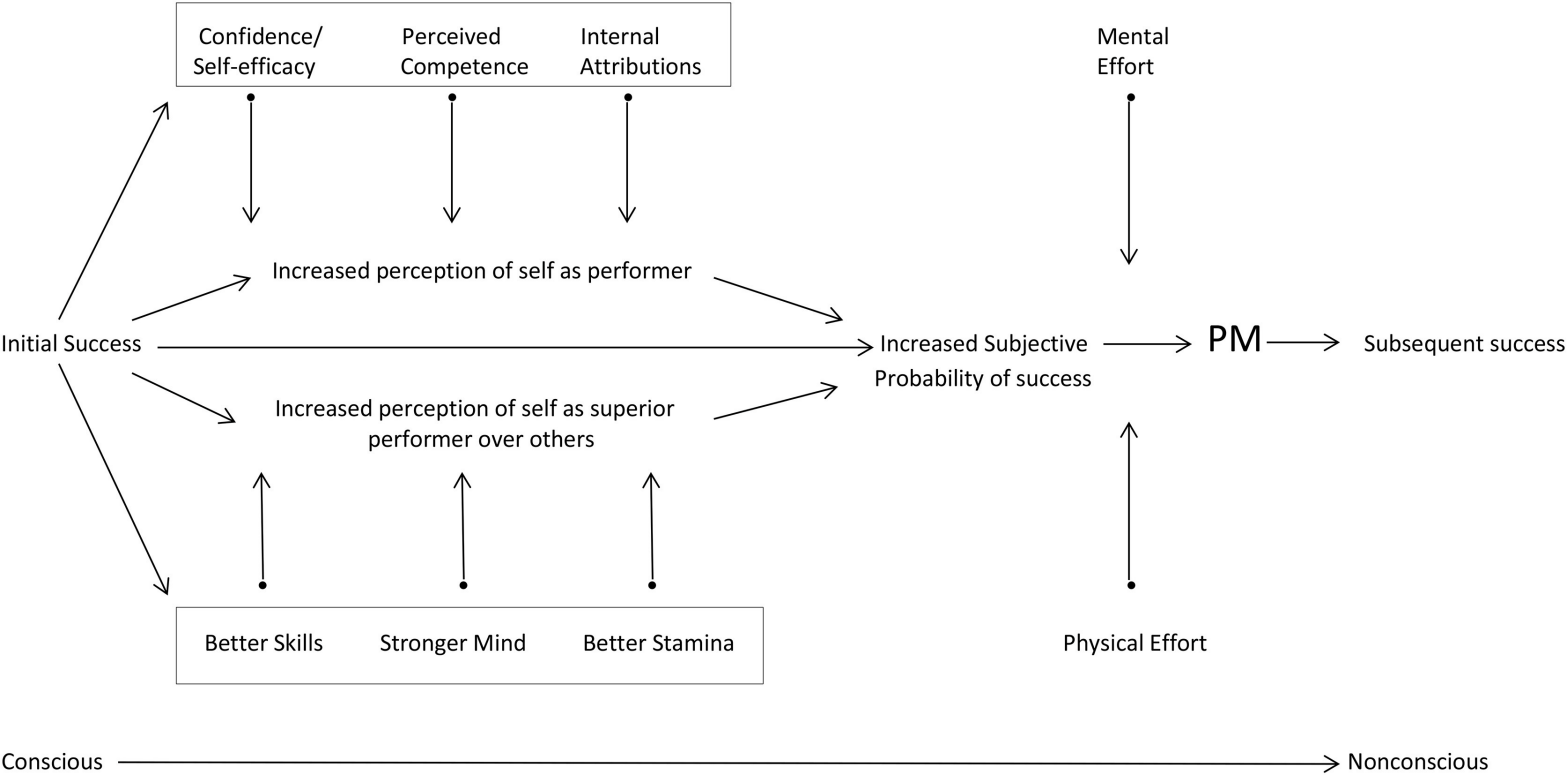
**Psychological momentum (PM) model for competitive situations**. Reprinted with permission and adapted from Iso-Ahola and Dotson (2015).

In general, people strive to be efficient in completing tasks and in doing so, to save time and energy. PM facilitates this efficiency by making successful task completion more likely and faster. This *efficiency principle of PM* means that whatever tasks people undertake, perceptions of positive PM enhance their sense of success in goal pursuit. When they initially experience success, their self-confidence and competence grow, leading to heightened expectations, expanded mental and physical effort in task performance, increased perceptions of positive PM, and a greater likelihood of success (Figure 1; Iso-Ahola and Dotson, 2015).

A corollary of the efficiency principle of PM is that people do not want to be interrupted in their task performance when PM is experienced. Interruptions mean that performance has to be started again from scratch, which naturally slows performance or thwarts it altogether, with such perturbations increasing energy expenditure. In Division I intercollegiate basketball games, for example, task performance was reduced by 56% when the opposing team took a time-out in efforts to interrupt the competitor's PM of successful performances (Mace et al., 1992). Interruption of PM likely changes performers' perceptions of task difficulty and the work needed for success (Markman and Guenther, 2007), and is as disruptive to persons raking leaves in their backyard as it is devastating to elite athletes performing at stadia and short-term traders on Wall Street (Antonacci, 2014).

Having gained positive momentum, task performance is perceived as easier and smoother, but when the task has to be restarted it becomes more demanding and difficult, often leading to negative emotions. For example, when aggressive drivers cut off other drivers interrupting their momentum of smooth driving, tempers may flare resulting as much from the lost perceived momentum as from anger at other people not following the rules. More generally, such “rubbernecking” of continuously gaining and losing positive momentum, be it in traffic or other domains of human performance, is frustrating and energy-consuming for performers, resulting in deteriorated performance (e.g., reaction time). This is an important area for empirical research in the future.

## Initial success and PM

Where does it all start? PM has to be created, it just does not happen. Given that success (vs. failure) is one of the most powerful variables in all of psychology (Kluger and Denisi, 1996), it is not surprising that initial success becomes the critical factor in the birth of PM. Experiencing success instantly changes people's perceptions of themselves as performers (Feather, 1968), as well as those of their opponents (Figure 1). In general, however, success is likely to lead to subsequent or future success only if initial success gives rise to the psychological process of momentum. This, in turn, means that PM can be a mediating mechanism between early and subsequent success. Initial success increases performers' self-confidence and sense of competence and facilitates internal attributions to ability and skills (Feather, 1968; Iso-Ahola and Dotson, 2014). These psychological effects, however, must translate into an increased subjective probability of success for PM to be born and for the overall causal effect of past success on future success to occur (Rosenqvist and Nordstrom Skans, 2015).

If the situation involves a face-to-face competitor, initial success not only enhances one's perception of him/herself as a performer but simultaneously, his/her perception of the opponent: “Since I am more skillful, have a stronger mind and better physical condition, I can beat her.” Such perceptions in part enabled the unseeded Roberta Vinci to build PM and beat the world's number 1 ranked player, Serena Williams, in the 2015 U.S. Open semifinal match.

In competitive situations, the two overall perceptions (oneself as a performer and oneself in relation to the opponent) are the key. As Figure 1 shows, each of these two general perceptions in turn is underpinned by three “latent” factors or perceptions. If individuals see themselves as strong performers technically, physically, and mentally (confidence, competence, and attributions to ability), and simultaneously perceive themselves being superior over the opponent on the three factors (skills, mind, and stamina), their subjective probability of success grows appreciably. This altered state of mind not only makes PM possible but likely.

Although many performance situations do not involve competitors, the same psychological process applies to such settings as well. For example, if people experience PM when raking leaves, they may have confidence that they can finish the whole backyard, saying to themselves, “I am on a roll, I have completed 2/3 of the raking and have enough strength to complete the task.” Thus, the perception that success is possible is the critical determinant and consequence of PM. When people sense that they can succeed (i.e., perceived likelihood of success is increased), they expand their mental (e.g., concentration) and physical effort (Figure 1), which leads to a positive-upward-feedback spiral of more PM and more success (Feather, 1968; Kluger and Denisi, 1996; Iso-Ahola and Dotson, 2014; Hubbard, 2015a; Rosenqvist and Nordstrom Skans, 2015). Finally, it should be noted that PM can occur between and within tasks and performances; in tennis, for example, from one game to another or from one match to the next (between), or from one volley to another (within). Since PM is short-lived in general, it is more likely that the PM effects materialize more readily within rather than between performances, but this remains to be investigated.

## Key mechanism

We have previously theorized that momentum effects are manifested in three specific patterns of performance outcomes (Iso-Ahola and Dotson, 2014): (1) an individual or team that has accumulated more occurrences of momentum during a contest or task performance (frequency effect), (2) whose occurrences of momentum last longer (duration effect), and (3) whose occurrences of momentum are of greater intensity (intensity effect), has a greater likelihood of succeeding. These effects culminate in the individual performance such that a performer who is higher in any single effect (frequency, duration, intensity), or a combination of the three, is more likely to succeed. The effects are underpinned by a sense of confidence and competence and internal attributions, as well as perceptions of superiority over an opponent (Figure 1). In other words, the frequency, duration, and intensity effects of PM do not materialize without these perceptions.

The three effects are manifestations of the earlier-stated efficiency principle of PM that people strive to be efficient in completing tasks. For example, it is not efficient if one continuously has to stop and restart his/her performance; such perturbations will be manifested in the reduced duration effect. The longer the momentum lasts, the more efficient and better is performance. It follows that the antecedents of PM (Figure 1) are also antecedents of the efficiency of performance.

**Figure 2:**
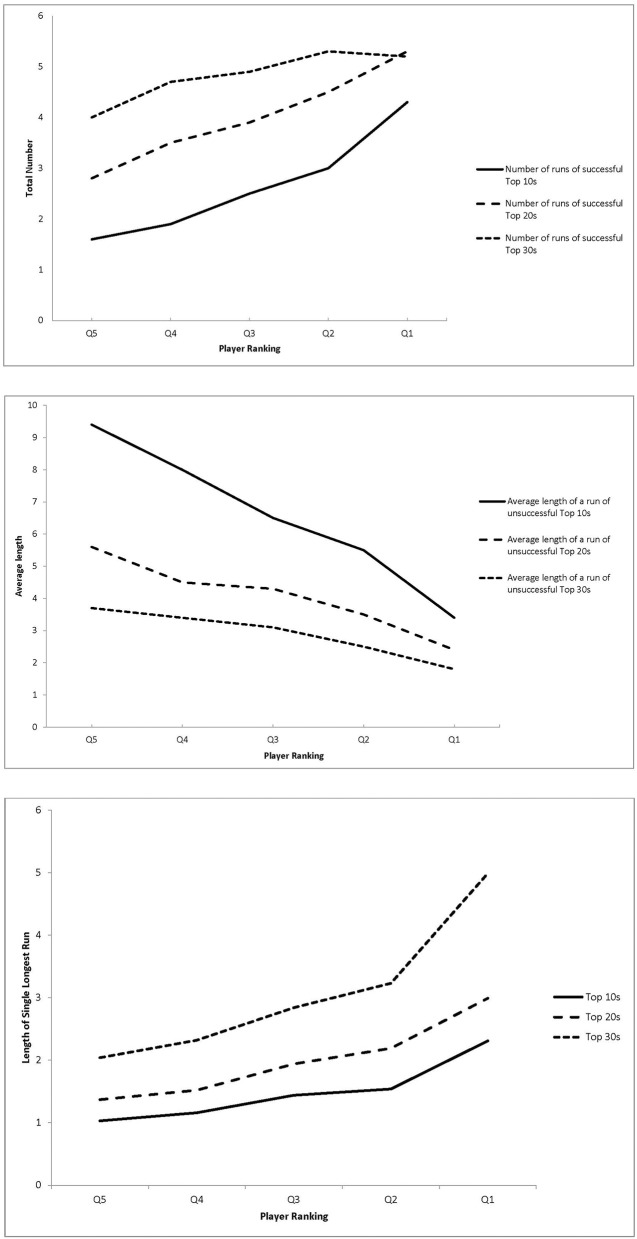
**Number of runs of successful top performances as a function of player ranking (left panel); average length of a run of unsuccessful top performances as a function of player ranking (middle panel); length of the single longest run of successful top performances as a function of player ranking (right panel)**. Reprinted with permission and adapted from Iso-Ahola and Dotson (2015).

In one study, these effects were tested using more than 11,000 tournament outcomes over 4 years of competition on Professional Golf Association's (PGA) tour events (Iso-Ahola and Dotson, 2015). Compared to lower-ranked players, better performers generated more momentum runs, made them last longer, and bounced back faster from their failures (i.e., shorter runs of unsuccessful performances; Figure 2). Such effects were more evident for high intensity successes (i.e., Top 10 achievements). Further, the number of successful runs and their length explained 91.5% of variance in Top 10 achievements, 87.1% in Top 20s, and 84.9% in Top 30s. To see this momentum effect in another way, we removed the number of runs and their length from regression analysis and computed the adjusted eta squares. As a result, eta squares were reduced to trivial effect sizes: Top 10 (to 0.016 from 0.58), Top 20 (to 0.015 from 0.49), Top 30 (to 0.009 from 0.44), and cuts made (to 0.007 from 0.13). In short, the number of successful runs and their duration powerfully explained differences in performance outcomes and were particularly evident in high-intensity achievements (Top 10s).

Other related tests of the momentum effects have been reported in numerous studies, mostly in individual sports, especially those in which players feel greater control over their performance (Oskarsson et al., 2009), but also in teams sports (e.g., Raab et al., 2012), as well as in mutual fund investing (e.g., Hendricks et al., 1993; for a comprehensive review of research, see Hubbard, 2015a,b). Taken together, the empirical results suggest that PM is a central ingredient of performance and provides a good explanation of when and why performance leads to success.

### “Ability” vs. PM

A counter argument has been made that physical “ability” itself can explain the difference between successful and unsuccessful performances. There are, however, several theoretically and empirically-based reasons to reject such a competing argument. If “ability” were the decisive factor, the unseeded player who had lost all previous matches to Serena Williams would not have been able to beat her in the U.S. Open semifinal match. By definition, ability is fixed whereas “skills” fluctuate with situations. Thus, it is a question of how competitors take advantage of their own and opponents' changing skills in a contest; this is mainly achieved by psychological means because there is little variance in requisite physical and technical skills among competitors. It is well-known that as ability/skill increases, intra-performer and inter-performer variance decreases. The higher the level of competition, the less *absolute* measures of skill (e.g., service speed) produce variance within and between players. As inter-individual variance is reduced with increased practice in motor tasks, performers become more alike with increasing skills (Ackerman, 2007), thereby making psychological factors (e.g., PM) main determinants of performance. It is then not surprising that PM explains most of the variance in performance at the highest level (i.e., pro tennis; Jackson and Mosurski, 1997).

“Underdogs” win because of newly found psychological resources, particularly PM, not because of a sudden increase in fixed ability. In zero-sum games, this means that winning underdogs have positive momentum (i.e., more frequent and more lasting occurrences of momentum) while their opponents simultaneously have negative momentum, with the net result of enhanced performance in the former and deteriorated performance in the latter. Both success and failure, especially when they develop into streaks, have powerful psychological effects (Kluger and Denisi, 1996). Thus, it is likely that the underdog's (Vinci's) early and subsequently cumulating successes created positive PM for her and simultaneously negative PM for Williams. Through her winning performance, Vinci demonstrated not only her possession of equal technical skills but also, utilization of psychological processes to her advantage (Figure 1), as also echoed by Williams' comment: “She played out of her mind.” In short, a winning performance is a function of both physical and mental skills, but the latter become a more critical determinant of success because of lesser variation in physical than mental skills.

If “ability” were the only or even critical factor, elite performers would never “choke” (e.g., Baumeister, 1984; Gray, 2004; DeCaro et al., 2011), nor would their performance be enhanced by psychological factors. Yet, empirical research has repeatedly shown that the effects of various social psychological factors occur over and above the effects of so-called “ability” in laboratory tasks such as ability to solve anagrams (e.g., Feather and Saville, 1967; Feather, 1968), as well as in elite performance in international competition (Marsh and Perry, 2005). Also, while the PM effect occurs at different skill levels, its importance grows with increased skills further challenging the “ability” explanation (e.g., Iso-Ahola and Mobily, 1980). This is consistent with our earlier-described longitudinal data according to which effect sizes were reduced to almost nothing when the momentum effects were removed from the statistical analysis, especially at higher levels of performance.

In the most direct test of the role of ability vs. PM in performance outcomes, Jackson and Mosurski (1997) compared the power of four models based on 2 years of data on elite players: (1) simple Independence between present and past performance, (2) PM, (3) Independence with a normal random effect (daily fluctuation in players' ability), and (4) PM with a normal random effect. Results showed strongly that PM was the best and Independence the worst model to explain performance success, and the addition of day-to-day variation in player ability contributed very little to the overall explanation, thereby ruling out the random effect (fluctuation in “ability”) and refuting the claim that PM or “hot hand” does not exist (Gilovich et al., 1985).

Finally, it should be noted that “ability” does not exist as an independent entity but mostly manifests itself as a product of “deliberate” practice. In other words, deliberate practice turns initial ability into physical and technical skills. In general, deliberate practice accounts for about 50% of the total variance in various domains of human performance (Ericsson and Ward, 2007). In contrast, for example, the working-memory related ability to sight-read (to play music with little or no preparation) was found to explain only 7.4 % and deliberate practice 45.1% of the performance variance in individuals with a median of 4160 h of cumulative deliberate practice (Meinz and Hambrick, 2010). Increased skills obtained through deliberate practice allow performers to focus on other relevant factors (e.g., strategy) and therefore take advantage of the power of psychological processes, specifically PM.

These findings are also important in showing that there is a ceiling to how much of the total variance deliberate practice and ability can explain in various domains of human performance. If their combined effect in a given domain is about 50%, most of the variance of the other half comes from the contribution of psychological factors. How much of that percentage in turn is attributable to PM is unknown at the present. But as Jackson and Mosurski's data (1997) showed, it certainly is much more than that of daily fluctuations in performers' ability.

Taken together, both theoretical and empirical literatures suggest that the so-called ability is a relatively poor discriminator between successful and unsuccessful performances and performers. Instead, psychological processes play a more critical role at increasing levels of skilled performance; of these processes, PM is one of the most important. PM's importance is seen in many ways, from motivational effects to enhancement of concentration to facilitation of the transition from controlled to automatic processing, as will be discussed next.

## Future theory and research: from conscious to nonconscious

In general, with increasing skill, human performance advances from conscious to nonconscious or automatic execution of movement (Fitts and Posner, 1967), from controlled to automatic processing of information (Schneider and Shiffrin, 1977; Shiffrin and Schneider, 1977). Automatic processing, however, is not chipped in stone; it can easily be interrupted, for example, by pressure (e.g., Gray, 2011). But it can also be enhanced, especially by PM, as we suggest here. When initial success changes individuals' perceptions of themselves as performers and their expectations of success (Figure 1), PM becomes a psychological and largely conscious experience of mental strength and a resource that is needed for success (e.g., Shaw et al., 1992). With well-practiced skills, especially in high-level performers, we suggest, the psychological experience resulting from initial success quickly turns into PM that facilitates automatic processing, leading to improved performance independent of attention and effort put into controlled processing (Shiffrin and Schneider, 1977, p. 183).

Facilitation of automatic processing is reflected in greater neural efficiency (Yarrow et al., 2009), reduction of neural variability in task-relevant components *prior to* performance execution (Churchland et al., 2006), and “unfreezing degrees of freedom” of movements (Turvey et al., 1982)^1^. As expert performers are able to extract important stimulus information earlier than non-experts (Yarrow et al., 2009), nonconscious processes are likely to become more dominant and faster with increased experience and expertise. Thus, PM can help performers take advantage of the effect of earlier intensive practice to organize and coordinate neuron networks' activity (i.e., neuron spike synchrony, Kilavik et al., 2009), allowing neurons to integrate information over space and time (Dehaene, 2014), to optimize behavior and performance.

Evidence supports the idea that psychological and neurocognitive factors interactively determine success, as it has been found that early winning improves competitors' mindset (e.g., confidence) and adaptive cortical dynamics (i.e., reduced cognitive load related to working memory and greater engagement of task-relevant attentional processes; Hunt et al., 2013). Within a given performance situation, according to the mediational model of PM (Iso-Ahola and Dotson, 2014), a performer moves onward from predominantly conscious influences of initial success to PM that is experienced nonconsciously in the automatic execution of skills (Figure 1). That is, initial success first generates conscious psychological effects on performers, but the resultant PM enables them to rely more, if not exclusively, on nonconscious automatic processes, particularly in fast-paced situations such as basketball games. However, the process should not depend on a type of task as the mediational model of PM makes the same prediction for more cognition-based tasks (e.g., stock trading, common household activities) as well. Empirical research, though, is needed to test the veracity of this prediction.

As a whole, PM is largely a nonconscious force but underpinned and produced by the assistance of the two overall perceptions and their underlying “latent” factors (Figure 1) that are mostly experienced consciously. Although PM's effects on performance are primarily nonconscious, the process leading to PM's birth involves the interplay of both conscious and nonconscious operations of the human mind.

Thus, an important area for future research is not only PM's facilitation of automatic processing and diminishment of interfering effects of conscious processing but also, how the two processes work seamlessly together to enable high-level performance. The interplay between conscious and nonconscious processes is evident when PM is perceptually lost and in the worst case, allowed to turn into negative PM. In this situation, performers relegate the guiding and enabling power of nonconscious processing back to their conscious mind as they start losing confidence in themselves as performers and thus begin consciously steering once-automatic movements, increasing the likelihood of serious negative consequences. In general, conscious monitoring of skills is detrimental to performance (Gray, 2004). To prevent it, it is therefore essential that positive PM be maintained as long as possible (i.e., “duration effect”). The power of positive PM may lie not only in its ability to consciously energize performers but, when becoming a nonconscious process, to help them focus on the automatic execution of the task and avoid distractors. In this sense, PM becomes a countervailing force to “choking.” These ideas, however, await empirical testing.

## Author contributions

Both authors have made substantial and direct contributions to the work, and approved for publication.

### Conflict of interest statement

The authors declare that the research was conducted in the absence of any commercial or financial relationships that could be construed as a potential conflict of interest.

## Footnotes

^1^Neural efficiency refers to efficiency of cerebral-cortical processing and is exhibited to a greater extent by expert performers compared to novices. Task-relevant factors are those that are critical for performance (e.g., focusing on the task at hand), whereas task-irrelevant factors are distractors. “Unfreezing of degrees of freedom” refers to a greater number and use of non-rigid and efficient movements in motor performance.
